# Integration of Psychiatric Advance Directives Into the Patient-Accessible Electronic Health Record: Exploring the Promise and Limitations

**DOI:** 10.2196/68549

**Published:** 2025-03-18

**Authors:** Julian Schwarz, Eva Meier-Diedrich, Matthé Scholten, Lucy Stephenson, John Torous, Florian Wurster, Charlotte Blease

**Affiliations:** 1 Center for Mental Health, Department of Psychiatry and Psychotherapy Immanuel Hospital Rüdersdorf Brandenburg Medical School Theodor Fontane Rüdersdorf Germany; 2 Faculty of Health Sciences Brandenburg Brandenburg Medical School Theodor Fontane Neuruppin Germany; 3 Institute for Medical Ethics and History of Medicine Ruhr University Bochum Bochum Germany; 4 South London and Maudsley NHS Foundation Trust London United Kingdom; 5 Division of Digital Psychiatry Beth Israel Deaconess Medical Center Harvard Medical School Boston, MA United States; 6 Chair of Quality Development and Evaluation in Rehabilitation Institute of Medical Sociology Health Services Research, and Rehabilitation Science University of Cologne Cologne Germany; 7 Faculty of Human Sciences & Faculty of Medicine University Hospital Cologne Cologne Germany; 8 Department of Women’s and Children’s Health Uppsala University Uppsala Sweden

**Keywords:** advance statements, advance choice documents, advance care planning, mental health, online record access, patient accessible electronic health records, interoperability, fast healthcare interoperability eesources, FHIR, self-binding directives, mobile phone

## Abstract

Psychiatric advance directives (PAD), also known as advance statements or advance choice documents, are legal documents that enable people with mental health conditions to specify their treatment preferences in advance for possible future crises. Subtypes of PADs include crisis cards, joint crisis plans, and self-binding directives (also known as Ulysses contracts). These instruments are intended to improve service user involvement and need orientation in the care of mental crises and to avoid traumatization through unwanted treatment. The existing evidence suggests that people who complete a PAD tend to work more cooperatively with their clinician and experience fewer involuntary hospital admissions. Nevertheless, PADs have not been successfully mainstreamed into care due to multiple barriers to the implementation of PADs, mainly around the completion of PADs and their accessibility and use in crises. The reasons for this include the lack of support in the completion process and acceptance problems, especially on the part of professionals. The research to date primarily recommends support for service users from facilitators, such as peer support workers, and training for all stakeholders. In this article, we argue that while these approaches can help to solve completion and acceptance challenges, they are not sufficient to ensure access to PADs in crises. To ensure accessibility, we propose digital PADs, which offer considerable potential for overcoming these aforementioned barriers. Embedded in national health data infrastructures, PADs could be completed and accessed by service users themselves, possibly with the support of facilitators, and retrieved by any clinic in an emergency. We highlight the strengths and limitations of digital PADs and point out that the proposed solutions must be developed collaboratively and take into account digital inequalities to be effective support for people with serious mental health conditions.

## Introduction

In recent decades, interest in self-determination in health care and service user autonomy has grown considerably, as evidenced by the increasing implementation of interventions to promote shared decision-making and advance care planning in health care [1,2]. In the field of mental health, this is a contested issue in view of perceived potential conflicts between the autonomy and well-being of service users. Although extensive attempts have been made for decades to reduce involuntary interventions in mental health care, this has only been successful to a limited extent [3].

A key intervention in this context is advance care planning using a so-called psychiatric advance directive (PAD), also known as an advance statement or advance choice document. PADs are legal documents that allow individuals to determine in advance how they would like to be treated in the event of a future mental health crisis or psychiatric emergency [4-6]. Subtypes of PADs include crisis cards, joint crisis plans, and self-binding directives (also known as Ulysses contracts or arrangements) [7]. Self-binding PADs are distinctive in that they contain a clause in which service users can request future involuntary hospital admission and treatment during a mental health crisis whilst recognizing that at the time these interventions are required, they are likely to refuse them [8,9]. Despite significant differences between the variants of PADs, they aim to help mental health service users ensure that their treatment wishes are heard and acted upon, particularly in situations where they are unable to express them or lack the threshold capacity to make decisions. To facilitate understanding and reduce complexity, the various forms of advance care planning in mental health care are summarized under the term PAD, unless they are specifically mentioned.

A growing body of scientific papers suggests that PADs can increase the autonomy and perceived control of service users [4,10], strengthen the therapeutic alliance and patient-physician communication [4,6,11], improve adherence [12,13], reduce coercive measures and involuntary admissions [14-17], and could possibly lead to better overall treatment outcomes [12]. In addition, various bodies of the United Nations have stated that the Convention on the Rights of Persons with Disabilities demands the implementation of PADs [18]. Against this backdrop, frameworks to promote PADs for mental health crises have been and are being created in more and more countries. For example, the UK government has enacted legislation for PADs in England and Wales and is currently evaluating its implementation [19]. The Mental Health and Wellbeing Act in Australia and Washington State’s SB 5660 in the United States are intended to anchor PADs more firmly in care [4,20,21]. In Germany, PADs are legally binding under the Civil Code and the country’s clinical guidelines for the prevention of coercion recommend the implementation of PADs with the highest level of evidence [22-24]. Nevertheless, the available evidence on PADs is considered to be expandable, partly due to the comparatively small sample sizes in some cases and the still small proportion of studies on the topic conducted with service users.

Despite the benefits of PADs for service users, numerous challenges remain in their practical application [14,25]. In this viewpoint paper, we identify outstanding difficulties with PADs, such as the documents not being completed, not being accessible in crises (eg, because people do not carry them with them or they are not accessible in the treating clinic), clinicians ignoring PADs, PADs not being kept up to date, or the existence of conflicting versions of a PAD (eg, because paper and pencil updates were not distributed among all parties involved) [26]. To address these problems, recent reviews suggest improving PAD implementation strategies through measures such as training for professionals, service users, and their relatives [8,14]. We consider these measures useful but argue that their impact may be limited if PADs are not accessible to all stakeholders at all times in an up-to-date form. As a solution, we propose digital PADs embedded in electronic health records and show that they may provide longer-term tangible outcomes and increase the usability, practicality, and relevance of PADs, which in turn would have a positive impact on their utility and clinical impact. We explore the strengths and limitations of digital PADs and emphasize that for the innovation to work, it is essential that the PADs are interoperable, ideally integrated into each country’s health care data infrastructure via Fast Healthcare Interoperability Resources. Interoperable means in this context that the content of the PAD is stored in a digital standardized form, namely via Fast Healthcare Interoperability Resources, which permits, for example, smooth access by the different medical information systems of various health care institutions.

## Current Challenges With PADs in Crisis Care

Many studies have explored the benefits and challenges of implementing PADs [4,25,27-29]. We will focus on these challenges below, as these are the key starting points for the proposed solution.

### Problem With Drafting PADs

In a review of service users’ perspectives on PADs, Braun et al [14] found that in 73% (30/41) of the studies, completing a PAD was a challenge for those involved. Service users reported a lack of knowledge and understanding of how PADs work [5,30-32]: they often do not know what to write in a PAD, and the templates provided are often without sufficient explanations and examples, making it challenging to complete them [6,33]. In addition, some service users find completing a PAD too complicated or time-consuming [34-36]. Doubts about one’s own decision-making ability at the time of preparation also discourage service users [6,27,31,37]. Finally, the creation process can be emotionally distressing, especially if it evokes memories of previous involuntary treatment [11,36,38,39].

Another difficulty in creating a PAD is the lack of support. For example, many service users do not have a trusted person to support them [11,36,38]. Some service users are also concerned that potential facilitators, such as the treating psychiatrist or relatives, could exert undue influence on the contents of the PAD by introducing their own potentially divergent preferences [35,39]. In addition, clinicians usually have little or no financial or time resources to draft a PAD together with the service users or even to advise them [40,41]. Professionals are reluctant to invest time in creating PADs [42]. It is, therefore, not surprising that service users report unclear or inadequate instructions and insufficient follow-up by professionals in the event of any queries [30,43].

Overall, these findings illustrate that service users need clear guidance and support to create an actionable PAD that meets their needs. Professionals should be given the necessary time to support the completion.

### Problems With Accessing PADs

A key problem is that PADs are often not stored so that they are up-to-date and accessible to all relevant stakeholders during a crisis [14,27,37,41,44]. Reasons for this are that those primarily involved in the use of a PAD, such as staff in the emergency department, inpatient wards of a hospital, judges, guardians ad litem, and legal guardians often do not know whether a PAD is present or not [25,28,45], that service users cannot provide information about the presence of a PAD in the event of a crisis, that professionals are not made aware of the presence of a PAD through a corresponding notification in the hospital information system, that the PAD is not available in digitized form, or that possible standard operating procedures for crisis and de-escalation management in a hospital are either not available or do not take PADs into account. Various studies show that these considerable access problems lead to uncertainty and irritation among service users, as an exemplary statement of a service user from a qualitative focus group study shows:

I wasn’t at the stage of going into hospital, but I’d been on a high and they were a little bit worried. And what concerns me was that they didn’t have a copy of your crisis plan [26].

The barriers to access are even higher when service users present at a hospital where they have not been treated previously [25]. In such situations, it is unknown what kind of care and medication was helpful in previous crises, nor can previous doctors’ letters or PADs be accessed unless the person concerned brings these with them themselves. Particularly in the case of involuntary hospital admission or treatment or use of coercive measures, a lack of knowledge about individualized crisis treatment can lead to inadequate treatment, harm, and traumatization [46,47].

### Problems With Applying PADs

Even if it is ensured that a PAD is accessible, this does not mean that it is actually taken into account in crisis management. For example, about 90% (35/41) of the studies identified in the review by Braun et al [14] that examined the service user perspective on PADs found evidence of application problems. An important factor here is that PADs are not followed in practice by professionals (approximately 61% of studies; 25/41) for various reasons. Service users reported that PADs were not endorsed by professionals in principle (approximately 17% of studies; 7/41) or PADs are not legally binding and hence could only be applied to a limited extent (approximately 22% of studies; 9/41). Another reason for not using PADs is the fear among professionals and some service users that the contents of PADs may be outdated because they are not regularly updated and therefore no longer reflect the current preferences of service users or current treatment guidelines (approximately 12% of studies; 5/41). In this context, community stakeholders expressed concern that service users would not take responsibility for keeping their PAD up to date over time [48]. Conversely, service users experienced that PADs were only initiated by professionals but not followed up, let alone integrated into outpatient treatment in the longer term, for example, through an accompanying regular exchange about the PAD [38].

In addition, the problem remains that not all contingencies of a possible future mental health crisis can be covered by a PAD [8,37,41]. Accordingly, stakeholders primarily criticize the lack of flexibility of PADs and would like them to be formulated as concretely and yet as widely applicable as possible so that they can be applied to a wide variety of mental health crisis scenarios [37]. This challenge is particularly relevant for self-binding PADs, as they can also be enforced over the dissent of a service user who lacks decision-making ability and against potentially divergent treatment recommendations from clinicians [8].

Finally, a lack of knowledge and inadequate training of clinicians in the use of PADs in emergency situations hinders their application. It is often unclear when a PAD is read by which member of the mental health team, how it is implemented, and who takes responsibility for this [49].

## Currently Proposed Solutions

A number of measures are described in the literature, all of which aim to improve the implementation of PADs.

The most promising of these is the use of human support, or “facilitators,” to guide the preparation of a PAD, which can significantly improve completion rates [50,51]. The role of facilitator can be assumed by professionals, peer support workers, patient advocates, or legal representatives. A recent RCT study from France shows that peer support workers-facilitated PADs can reduce the number of compulsory admissions compared to conventional PADs [52]. This might be explained by the fact that those affected can open up about this sensitive topic more confidently with a person with personal experience of mental distress or even involuntary treatment [53] than possibly their relatives or even practitioners, who may have different interests in crisis treatment than the affected person themselves. On the other hand, the use of facilitators could make the intervention more complex and time-consuming, as a considerable amount of additional training and personnel is required.

Alternatively, the literature recommends offering financial incentives, particularly for clinicians, to compensate for the additional time spent on supporting service users to complete PADs [22], or providing user-friendly templates and clear instructions for completing them—both of which have seen limited implementation in practice so far [41,54]. Furthermore, research suggests specific groups that could be targeted to increase the likelihood of PAD completion, such as those with strong social integration, those who have had negative treatment experiences in the past, and those who have recently experienced an acute mental health crisis [14]. It also suggests there are specific groups who might find it more challenging to complete PADs but are more likely to benefit from them. For example, service users from Black communities have identified additional barriers to completion, but research suggests additional benefits if service users are supported to overcome these hurdles [55].

Finally, various education and training interventions for service users and clinicians are recommended to offer more knowledge about PADs and the associated legal and ethical aspects to reduce misunderstandings and fears and increase uptake [56-58]. Training should also help to positively influence the aforementioned barriers to access, especially those aimed explicitly at ER personnel or rather detailing the use of PADs in emergency situations [25,49]. At the same time, it is important to emphasize that the existing evidence suggests that these measures are only effective to a limited extent and that administrative staff often have more knowledge about PADs than service users themselves [49,59]. Shields et al [25] therefore recommend integrating training into both undergraduate education and continuing professional development.

Overall, the currently proposed solutions can potentially promote the completion of PADs and, in principle, reduce involuntary interventions. Nevertheless, the problem remains that neither PADs nor facilitation is widely implemented in practice [15,16,51]. In addition, many trivial access problems remain unresolved, such as ensuring that the relevant stakeholders always have access to the same, up-to-date PAD version.

### Integration of PADs Into the Electronic Health Record

The idea of making the PAD accessible in digital form is not new, and the following is a brief overview of efforts in this regard in various countries. As early as 2001, a US study concluded [60] that “without a computerized system for storing and retrieving service users’ PAD information, preparing a PAD may do little more than the act of scrawling “help” on a scrap of paper, stuffing it into a bottle, and hurling it into the ocean.” Nevertheless, there are still only a few successful implementations of digital PADs in psychiatry. The situation is different in physical medicine, especially in palliative care, where there are already several digital applications for advanced care planning. In the United Kingdom, for example, “coordinate my care” has been implemented, which enables digital creation and access to advanced choice documents. A recent evaluation of this service shows that it is also used by mental health service users and their clinicians specifically for advance care planning of mental health conditions [61], presumably also due to the lack of such tools in the mental health field.

In the field of psychiatry, Murray and Wortzel [4] report that some states in the United States are already using so-called “online advance directive registries” in both medical and psychiatric care. Comprehensive descriptions and scientific evaluations of these approaches are not yet available. In addition, the so-called “My Mental Health Crisis App” was published in 2020, by the SMI Adviser initiative, a project funded by the US Substance Abuse and Mental Health Services Administration. It enables those affected to create a PAD using step-by-step instructions and then share it digitally [62]. The app even considers the different legal regulations in the US states and the resulting differences in practice when creating PADs, such as the mandatory presence of witnesses or notarization. Finally, the PAD created using the app can be shared via a QR code. While this enables barrier-free access, it risks violating privacy if the QR code falls into the hands of unauthorized persons. We are not aware of any scientific evaluations of this offer either.

A similar but purely web-based solution is currently being piloted in Germany as part of a study [63,64], where the PAD is embedded in a digital environment regularly used by service users to test online record access. The document can only be accessed by persons who have been previously authorized by the service user, such as outpatient clinicians (general practitioners, psychiatrists, psychotherapists), legal guardians, and relatives, using their own account. In addition, staff of the mental health hospital in the respective catchment area in which the service user lives have access to the PAD. In routine care in Germany, there is currently only one type of emergency dataset stored on the physical health insurance card of those with statutory health insurance. This card can theoretically be read by any ambulance service, although it is unclear to what extent this function is currently being used.

The only known digital and interoperable implementation of PADs in the field of mental health is currently carried out in the United Kingdom, albeit only on an experimental basis, jointly by the NHS in coproduction with the startup Thalamos [26]. The initiative was prompted by recently introduced legislation that aims to address the increased number of involuntary psychiatric treatments in the United Kingdom in recent years, while also addressing significant failures to address racial inequalities in mental health care [65]. This is supported by findings that the significantly higher proportion of “Black Indigenous people of color” admitted under the law appears to benefit particularly from PADs [66,67]. Currently, a PAD culturally adapted for people from sub-Saharan Africa and the Caribbean is being piloted and stored in electronic patient records that are available to connected service providers such as acute hospital trusts and general practitioners [44,68].

### Strengths of Digital Interoperable PADs

We argue that the approaches to digitizing PADs presented so far have considerable potential to improve the care situation of those affected, especially when combined. This section describes the strengths of digital PADs, based on the vision of a comprehensive digital solution from the perspective of medical ethicists and clinical researchers, and explains how digital PADs can help to overcome the key challenges of paper-based PADs.

First, every service user diagnosed with a serious mental health condition, and especially those with a history of involuntary hospital admission and treatment, should be informed about the possibility of completing a digital PAD. This could be done in a variety of ways, both through the care system and through user-led services such as self-help groups. Research has shown that PADs are better accepted when the completion process is supported [4,25]. In addition to human support, a digital, AI-based assistant integrated into the PAD form interface could guide users through the completion process, as outlined by Redahan and Kelly [69]. It could provide explanations and example wordings and ensure that the document is completed fully and correctly, meaning it makes sense in terms of content. This would address a key problem, namely that service users often do not manage to complete the PAD without support [6,39,70]. One of the reasons for the limited uptake by service users may be that research into the implementation of PADs has been conducted primarily by clinical researchers and medical ethicists, including this article. To address the priorities and concerns of service users, and to develop a viable (digital) PAD that service users find helpful, it is critical to have a design process that systematically involves service users from the outset [71,72].

One of the strengths of digital PADs is that service users control PAD access. This means that they could determine and control which people, such as outpatient practitioners (general practitioners, psychiatrists, and psychologists), other people (friends, relatives, etc), or people from other institutions involved in involuntary treatment and hospitalization (judges, guardians ad litem, community mental health investigators, legal guardians), would have read or write access to their digital PAD. One exception could be psychiatric hospitals, whose staff should be able to access the digital PAD when the respective service user is admitted or when they present to the emergency room of a hospital, whereby each read access would be logged by name. This would be necessary to protect the highly sensitive personal data contained in the PAD and to ensure that PADs are only accessed by treatment providers in a psychiatric emergency. This in turn would require a clear (technical) role concept for all actors involved, defining who is allowed to see and process what [26].

A major challenge in the implementation of conventional PADs is the lack of time or the reluctance of clinicians to invest the necessary time [42]. If service users could prepare a digital PAD at their own pace with the help of the digital assistant as outlined above or with the involvement of a trusted person, the attending psychiatrist would only need to check the digital PAD for feasibility and make any necessary suggestions for adjustments during the consultation. This would significantly reduce the time required by clinicians.

One of the main problems with analog PADs was that different versions were stored in different places, which could lead to the PAD accessed in the acute situation not being up to date. Digital access enables the service users to keep the digital PAD up to date when preferences for emergency treatment change. At the same time, a notification system could be used to notify all stakeholders with access to the PAD of changes, for example, by email, making collaboration much easier.

Digital PADs could likely play a significant role in promoting service user involvement in decision-making in mental health care. Ideally, if digital PADs were official, standardized documents integrated into the national health data infrastructure of each country, they would be difficult for institutions or professionals to overlook. This approach would also help tackle the prevalent issue of staff’s lack of engagement with PADs [29].

### Limitations of Digital Interoperable PADs

Although the digitization of PADs has the potential to overcome many practical barriers, it also brings new challenges, particularly in terms of implementation, acceptance, and engagement.

The existing evidence suggests that people with serious mental health conditions tend to have less access to digital health services or use them to a limited extent [71-73]. In addition, they are less likely to have access to a (desktop) computer, the internet, or a modern smartphone [74,75], and more likely to have concerns about data security and to lack the necessary digital skills [76,77] to keep a digital PAD up to date. As such, digital PADs risk increasing existing inequalities unless these access barriers are specifically addressed [26]. The solution used should therefore be flexible enough to meet the needs of the heterogeneous target group of people with serious mental health conditions and to avoid digital exclusion. To increase the acceptance and reach of the intervention, a combination of high- and low-tech approaches may be useful. For example, the facilitator could store a service user’s treatment preferences in the digital PAD on the service user’s behalf and provide them with a printout. At the same time, it should also be possible to fill in only selected parts of the digital PAD, such as only a free text field on treatment preferences, as it may be that a highly structured template could be overly prescriptive for some service users and may not adequately reflect how they wish to convey their PAD in terms of content and format. Furthermore, those service users who require additional digital skills to set up a digital PAD, or who have privacy concerns, should receive appropriate training. The latter should also include professionals, as they often do not have the necessary skills either or do not know how to best integrate digital tools into treatment [78].

Another potential limitation concerns the support provided during the process of completing digital PADs. If the completion of a PAD is supported by chatbots or human, nonphysician facilitators, at least some of the exchange about how to deal with emergency situations is outsourced from the patient-physician relationship. This could lead to alienation between patients and practitioners, especially after involuntary treatment or coercion. One way to counteract this would be to use debriefings of involuntary measures to also discuss the PADs and to validate the extent to which the instructions contained in them were actually implemented. This review of the (non) application of the PAD, which could also be done online, should be attended by all key persons involved in the previous involuntary action, or at least the inpatient and outpatient treatment providers in addition to the service user concerned.

In addition, there are a number of barriers to paper and pencil PADs that are likely to apply to digital PADs as well. This concerns the fear, more pronounced among psychiatrists in particular, that there may be a “lack of quality information” in PADs or that “consumers desire to change their mind during crisis” [28]. Furthermore, there are concerns that “inappropriate treatment requests” could be included in the PADs, that these requests could entail risks, that there is a “lack of time to review the document,” and that additional documentation is required. These limitations can probably only be reduced by providing more training and information to all parties involved, regardless of whether the intervention is digital or analog. In general, in addition to training measures, it is essential to promote the dissemination of (digital) PADs, be it through web-based multimedia resources, such as those realized in English-speaking countries, or through appropriate educational initiatives enshrined in law, in order to improve knowledge and awareness of PADs among the population, which includes both potential service users and professionals [79].

Another problem that is not solved by digitalization is that in various jurisdictions in some countries, such as the United States and Australia, PADs are not legally binding, and medical orders (eg for measures involving deprivation of liberty, issued by the acting physician during a psychiatric emergency) overrule PADs [25]. Conversely, where self-binding directives apply, it is often not regulated which PAD contents are legally binding and which are not and can therefore be overruled by professionals [8,37]. Consequently, digitalization should be accompanied by a revision of the legislation on the application of PADs and lead to more clarity about revocability and enforceability. In this context, for example, it would be important to clarify how to proceed if service users revoke the digital PAD on their cell phone in the context of an acute mental health crisis.

Another limitation, related to the existing acceptance issues, is that digitalization could render an already complex intervention “PAD” even more complex. In addition, problems related to internet connection and technology, system interoperability, data security, and data protection must be overcome [80]. For the innovation to succeed, an overarching implementation strategy is needed that permeates the macro, meso, and micro levels and requires the will as well as the necessary IT resources and infrastructure.

While digital PADs could provide greater convenience and ease in making advanced health care decisions, there is a potential risk that people with serious mental health conditions might update their directives during acute illness episodes, which could lead to problematic or unintended decisions. In addition, due to the digital nature of these directives, there may be privacy risks and concerns about unauthorized access. This could be the case if a service user shares the access or QR code with unknown people or in public places such as social networks, which could harm the service user. How these and related risks might be managed will require greater forethought and provisions to guide and protect service users.

## Future Research Directions

The introduction of digital PADs offers promising opportunities as well as new challenges that need to be further explored. Future research should focus on a participatory approach to the digitalization of PADs to ensure that the expectations and needs of all stakeholders, particularly service users, are adequately addressed to ensure the needs-based and practical nature of the solution to be developed. We argue that before measuring “hard” outcomes, such as the reduction of involuntary interventions and inpatient treatment days, which should of course be a longer-term goal, efforts must first be made that aim at a careful development and implementation of digital PADs.

First, the requirements and barriers to the implementation of digital PADs should be thoroughly researched. This must include developing strategies to reduce digital exclusion to ensure that the main target group of the intervention, people with serious mental health conditions, are not left behind. Furthermore, for a future-oriented embedding of the PAD in the health data infrastructure, transnational initiatives such as the European Health Data Space should also be considered. Finally, the legal and ethical implications of PADs must be investigated, especially in countries where PADs are not legally binding, to ensure that these digital tools improve, and do not hinder, the autonomy of the service user and the quality of care. Only when satisfactory acceptance by stakeholders can be demonstrated, as evidenced by a fully completed digital PAD and its accessibility in emergency situations, does it seem reasonable to investigate the abovementioned hard outcomes, for example, in the form of a randomized controlled trial. In this context, a comparison with the paper-and-pencil version of the PAD also seems important to demonstrate a possible differentiation and superiority of digital PADs.

## Conclusions

The integration of PADs into electronic health records represents an important step toward equality in mental and physical health care. At the same time, it is in line with the trend toward increasing digitalization of involuntary psychiatric admission processes [81,82], which holds significant potential in terms of transparency, speed, and equity. Digital PADs have the potential to fundamentally change psychiatric crisis care by addressing long-standing challenges of printed PADs such as accessibility, outdated information, and limited stakeholder engagement. Their success will depend on the careful development of implementation strategies, training of professionals, and the inclusion of stakeholders in the design and evaluation process. Although digital PADs can overcome many of the barriers associated with traditional PADs, they also introduce new complexities that must be addressed through ongoing research.

## Abbreviations

PAD: psychiatric advance directive
